# Synthesis and Gas Transport Properties of Addition Polynorbornene with Perfluorophenyl Side Groups

**DOI:** 10.3390/polym12061282

**Published:** 2020-06-03

**Authors:** Gleb O. Karpov, Ilya L. Borisov, Alexey V. Volkov, Eugene Sh. Finkelshtein, Maxim V. Bermeshev

**Affiliations:** A.V. Topchiev Institute of Petrochemical Synthesis of Russian Academy of Sciences, 29 Leninsky prospekt, 119991 Moscow, Russia; karpov@ips.ac.ru (G.O.K.); boril@ips.ac.ru (I.L.B.); avolkov@ips.ac.ru (A.V.V.); fin314@gmail.com (E.S.F.)

**Keywords:** norbornenes, addition polynorbornenes, gas permeability, membrane gas separation

## Abstract

Polynorbornenes represent a fruitful class of polymers for structure–property study. Recently, vinyl-addition polynorbornenes bearing side groups of different natures were observed to exhibit excellent gas permeation ability, along with attractive C_4_H_10_/CH_4_ and CO_2_/N_2_ separation selectivities. However, to date, the gas transport properties of fluorinated addition polynorbornenes have not been reported. Herein, we synthesized addition polynorbornene with fluoroorganic substituents and executed a study on the gas transport properties of the polymer for the first time. A norbornene-type monomer with a C_6_F_5_ group, 3-pentafluorophenyl-*exo*-tricyclononene-7, was successfully involved in addition polymerization, resulting in soluble, high-molecular-weight products obtained in good or high yields. By varying the monomer concentration and monomer/catalyst ratio, it was possible to reach M_w_ values of (2.93–4.35) × 10^5^. The molecular structure was confirmed by NMR and FTIR analysis. The contact angle with distilled water revealed the hydrophobic nature of the synthesized polymer as expected due to the presence of fluoroorganic side groups. A study of the permeability of various gases (He, H_2_, O_2_, N_2_, CO_2_, and CH_4_) through the prepared polymer disclosed a synergetic effect, which was achieved by the presence of both bulky perfluorinated side groups and rigid saturated main chains. Addition poly(3-pentafluorophenyl-*exo*-tricyclononene-7) was more permeable than its metathesis analogue by a factor of 7–21, or the similar polymer with flexible main chains, poly(pentafluorostyrene), in relation to the gases tested. Therefore, this investigation opens the door to fluorinated addition polynorbornenes as new potential polymeric materials for membrane gas separation.

## 1. Introduction

Membranes are widely used for the separation of various gas mixtures in different industrial processes such as air separation, acid gas removal, off-gas conditioning, or hydrocarbon separation [1,2,3,4]. The nature of the selective layer of the membrane plays a crucial role in its gas separation performance, and polymer-based materials are mainly considered for the fabrication of such membranes due to their good mechanical and film-forming properties and low cost. Despite the fact that a great variety of different polymeric materials have already been tested in terms of their gas transport properties [5,6,7,8,9], there is still the need for further optimization of gas transport performance of the membranes to meet ecological and other challenges, like carbon dioxide removal from flue gas or natural gas conditioning [1,8]. Polymers containing saturated main chains and inert side groups can be considered promising membrane materials due to their apt combination of gas permeability and selectivity, chemical resistance, and stability of properties under an extended time of use.

Polynorbornenes, synthesized via the addition mechanism of polymerization, possess rigid and saturated main chains [10,11]. Furthermore, it was shown [12] that polymer properties can be varied in a wide range by the introduction of side groups of different types. As a result, various addition polymers derived from norbornenes were successfully developed for applications such as adhesives [13], OLEDs [14,15,16], catalyst supports [17,18], membrane materials [9,19,20], gas storage materials [21], and dielectrics [22,23,24]. Different norbornene-based membrane materials possessing extremely high gas permeability accompanied by high C_4_H_10_/CH_4_ and CO_2_/N_2_ selectivities were reported [9,25,26,27,28,29,30]; in some cases, these polymers possess good long-term stability of their gas transport properties [25,29]. It was found that the most attractive sets of properties can be obtained by the introduction of trimethylsilyl, alkoxysilyl, siloxanyl, and alkyl side groups [9]. At the same time, it is well known that the incorporation of perfluoroalkyl and perfluoroaryl side substituents can also lead to the enhancement of gas permeability [31,32,33,34,35,36,37]. Moreover, the presence of fluoroorganic groups results in a substantial increase in the chemical and thermal stability of polymeric materials, thus making it promising to combine the advantages of both addition polynorbornenes and polymers with fluoroorganic substituents. To the best of our knowledge, the gas permeability of an addition polynorbornene with fluoroorganic side groups has not been studied before. Herein, for the first time, an addition polymer based on norbornene with a perfluorophenyl group was synthesized, and the gas transport properties of the prepared polymer were studied in detail. The results obtained were compared with previously described data for the isomeric metathesis polymer based on the same monomer structure.

## 2. Experimental Section

### 2.1. Materials

Tricyclohexylphosphine (PCy_3_), palladium(II) acetate (Pd(OAc)_2_), and solvents from Sigma-Aldrich (Moscow, Russia), along with Na^+^[B(3,5-(CF_3_)_2_C_6_H_5_)_4_]^−^ (NaBARF) from J&K Scientific (Beijing, China), were used in the current investigation. 3-Pentafluorophenyltricyclononene-7 was synthesized according to a procedure in the literature [34]. Pd(OAc)_2_, NaBARF, and tricyclohexylphosphine (PCy_3_) were applied without purification. Chloroform was dried over CaH_2_ and distilled under argon.

The syntheses were carried out under argon using the standard Schlenk technique or in MBraun Glovebox (Malsch, Germany) in the framework of RSF #17-73-104999. 3-Pentafluorophenyltricyclononene-7 was stored in an inert atmosphere.

### 2.2. Methods of Polymer Characterization

NMR spectra were recorded using a Bruker “Avance 600” spectrometer (Bremen, Germany) at 600 MHz (^1^H NMR) and 151 MHz (^13^C NMR) in CDCl_3_ solution. Chemical shifts (δ) are reported in parts per million (ppm) relative to a reference (residual CHCl_3_ signal). ^19^F NMR spectra were recorded using a Bruker “Avancer^TM^ DRX400” spectrometer (Bremen, Germany) at 376.5 MHz for ^19^F spectra using CDCl_3_ as a solvent.

The FTIR spectra were recorded on a HYPERION 2000 Bruker FTIR Microscope (Bremen, Germany) associated with an IFS-66 v/s Fourier spectrometer for ATR.

Calorimetric measurements were conducted using a “Mettler” TA-4000 differential scanning calorimeter (Giesen, Germany) at a heating rate of 20 °C/min under argon.

The molecular masses of polymers were estimated by means of gel permeation chromatography (GPC, Milford, MA, USA) using a “Water`s” high-pressure chromatograph with a refractometric detector and a Microgel mix 1–5 mcm, 500 mm × 7.7 mm Chropack column; the solvent was chloroform, the sample volume was 100 mkl, and the concentration was 1 mg/mL.

Wide-angle X-ray diffraction (WAXD) data were obtained using a two-coordinate AXS detector (Bruker, Bremen, Germany) and Cu Kα emission (wavelength of 0.154 nm).

### 2.3. Film Preparation

The polymer films for gas permeation measurements were prepared by casting from 5 wt% chloroform solution of the polymer. The solution was poured into a steel cylinder with a diameter of 7 cm and a stretched cellophane bottom. The solvent was allowed to evaporate slowly at room temperature to yield the desired polymer films. After the formation of the films, the cellophane was detached, and the films were dried under vacuum at room temperature to a constant weight. A thermal treatment was not applied. The thickness of the films formed was in the range of 100–120 µm. The properties of the obtained membranes were measured immediately after evacuation. The time of sample investigation was 2 days.

### 2.4. Measurements of Gas Transport Properties

The single-gas permeability, diffusion, and solubility coefficients were determined via the constant volume, variable pressure (“time-lag”) method according to the Daynes–Barrer technique using a “Helmholtz-Zentrum Geesthacht” precise unit mounted with a Baratron pressure sensor (MKS Instruments, Andover, MA, USA, accuracy 10^−7^ bar) at 30 °C in the framework of RSF #17-73-104999. The feed pressure was 0.8 bar for all the gases.

Gas transport through a polymer film is usually described by a three-stage dissolution–diffusion process, characterized by the absorption of gas at the polymer/gas interface from the feed side, followed by diffusion of dissolved molecules through the membrane and desorption of gas particles from the polymer/gas interface on the low-pressure side. In the trivial case (where the permeate pressure is insignificant compared to the feed pressure), the penetrating flow obeys Fick’s law and the permeability can be expressed as the product of the solubility and diffusion coefficients (Equation (1)):

(1) P=D·S,

where *D* is the diffusion coefficient (cm^2^/s) and *S* is the solubility coefficient (cm^3^ (STP)/(cm^3^∙bar)).

The following Equations (2)–(4) were used to determine the gas permeability (*P*), diffusion coefficient (*D*), solubility (*S*) coefficients, and ideal selectivity for pure gases (α_A/B_) [38]:

(2) $$P=\frac{V_{p}\cdot l}{M\cdot R\cdot T\cdot ∆t}\cdot \ln\frac{p_{f}-p_{p1}}{p_{f}-p_{p2}}.$$

(3) $$D=\frac{l^{2}}{6\theta }$$

α(A/B) = P(A)/P(B) = (D(A)/D(B))·(S(A)/S(B)) = α_D_·α_S_ (4)

where *V_p_* is the permeate volume, *l* is the membrane thickness, *M* is the membrane area, *R* is the gas constant, *p_f_* is the feed pressure (considered constant in the time range Δ*t*), *p_p*1*_* and *p_p*2*_* are the permeate pressures at time moments 1 and 2, Δ*t* is the time difference between two points (1 and 2) on the pressure curve, *θ* is the time lag, and indices A and B relate to the gases chosen for calculation of the permeability, diffusion, and solubility selectivities.

The time lag, *θ*, can be obtained by linear extrapolation of the steady-state pressure increase curve to the time axis or to the initial pressure.

Subsequently, the solubility coefficient can be obtained from the found permeability and diffusion coefficients via the equation (Equation (5))

(5) $$S=\frac{P}{D}$$

High-precision MKS Baratron pressure sensors, with a reaction speed of less than 0.05 s, allowed us to determine the time lag *θ* with an accuracy of 0.5 s [39]. Thus, *θ* can be determined with an accuracy of less than 10%. For experiments with *θ* of the order of seconds, the error does not exceed 5%. Moreover, most of the absolute values of the permeability and diffusion coefficient errors consist precisely of determination of the time lag *θ*. The uncertainty values for the permeability, diffusivity, and solubility results are 5%, 5%, and not more than 10% (δ(X_1_) = (∆X_2_/X_2_) + (∆X_3_/X_3_)), correspondingly.

### 2.5. Addition Polymerization of 3-Perfluorophenyltricyclononene-7

The following example is given for a monomer/Pd(OAc)_2_/NaBARF/PCy_3_ ratio of 1500:1:5:2.

The catalyst solution was prepared prior to the polymerization by mixing 2.0 × 10^−2^ M chloroform solution of PCy_3_ (0.35 mL, 7 × 10^−3^ mmol), 4.5 × 10^−3^ M chloroform solution of Pd(OAc)_2_ (0.78 mL, 3.5 × 10^−3^ mmol), and 2.0 × 10^−2^ M chloroform solution of NaBARF (0.87 mL, 1.74 × 10^−2^ mmol) and stirring for 5 min. The monomer (0.1 g, 0.35 mmol), dissolved in 0.10 mL of chloroform, was injected into a 4 mL vial. The polymerization was initiated by adding 0.13 mL of the catalyst solution, and the mixture was stirred for 10 min. The reaction mixture was allowed to sit for 24 h at 30 °C and was then precipitated into hexane. The hexane was removed, then the polymer was washed with 3 × 3 mL of hexane and dried in vacuum. The polymer was reprecipitated twice from its chloroform solution into hexane and dried in vacuum at 60 °C to a constant weight. Yield 41%. M_w_ = 4.1∙10^5^, M_w_/M_n_ = 3.6.

^1^H NMR (CDCl_3_, ppm): 4.50–0.50 (m, 11H).

^13^C NMR (CDCl_3_, ppm): 146.99–143.29, 140.76–135.75, 119.95–117.60, 115.81–112.80 (m, C_6_F_5_), 58.75–36.43, 33.68–23.89 (m, C(1)-C(9) from tricyclononane units).

^19^F NMR (CDCl_3_, ppm): −141.80−(−)143.52 (m), −157.50−(−)159.94 (m), −163.12−(−)164.30 (m).

## 3. Results and Discussion

### 3.1. Synthesis of the Polymer

**Figure 1:**
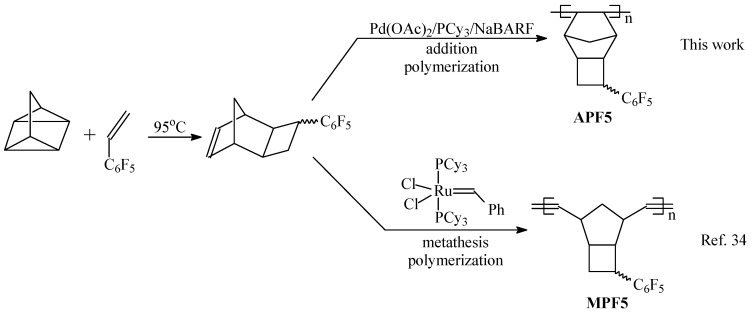
The synthesis of metathesis and addition polynorbornenes with C_6_F_5_ side groups.

**Figure 2:**
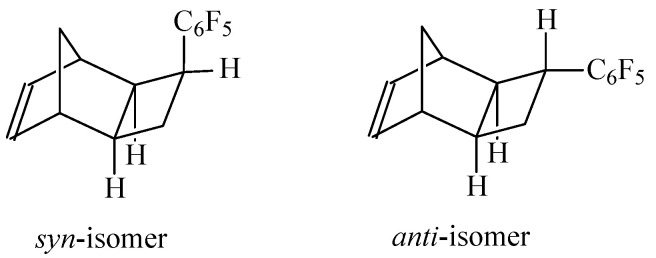
The structures of two isomers of 3-pentafluorophenyltricyclononene-7.

The desired norbornene-type monomer with C_6_F_5_ substituent was readily prepared by a [2+2+2]-cycloaddition reaction of quadricyclane and pentafluorophenylethylene (Figure 1) [34]. The isolated monomer was a pure *exo*-isomer of tricyclo [4.2.1.0^2,5^]non-7-ene and it consisted of two isomers (*syn*- and *anti*-isomers, Figure 2).

**Figure 3:**
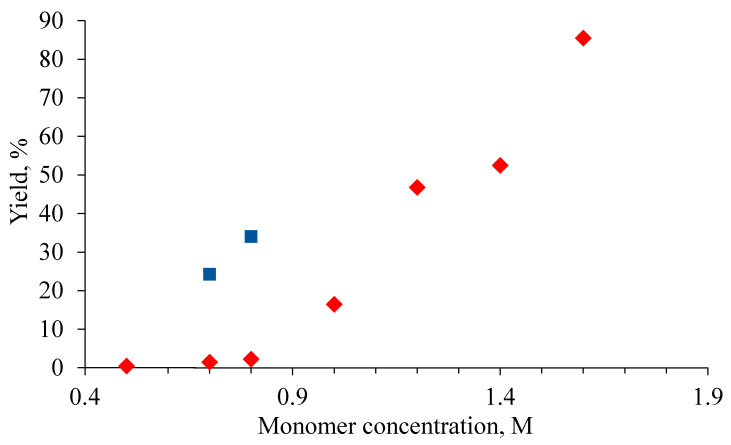
The influence of monomer concentration on the polymer yield of addition poly(3-pentafluorophenyltricyclononene-7) (the catalyst was Pd(OAc)_2_/NaBARF/PCy_3_; the Pd(OAc)_2_/NaBARF/PCy_3_ molar ratio was 1:5:2; the monomer/Pd molar ratio was 1000:1; the solvent was chloroform; the reaction time was 24 h (diamond markers) or 160 h (square markers); T = 30 °C).

Addition polymerization of the synthesized monomer was conducted in the presence of a three-component Pd system (acetate Pd(II) (Pd(OAc)_2_), tricyclohexylphosphine (PCy_3_), and Na^+^[B(3,5-(CF_3_)_2_C_6_H_5_)_4_]^−^ (NaBARF), Figure 1). This Pd-based catalytic system afforded a new vinyl(addition)-type polynorbornene in good or high yield depending on the polymerization conditions (Table 1 and Table 2). The yields of the polymer dramatically depended on the monomer concentration and the initial molar ratio of monomer to catalyst (Table 1 and Table 2, Figure 3). Thus, a decrease in the monomer/Pd molar ratio from 3000:1 to 500:1 led to a significant increase in polymer yield under the same polymerization conditions (Table 1).

In regard to the influence of the initial monomer concentration on the polymer yield, it seems that there is a certain threshold concentration for the polymerization to proceed efficiently (Figure 3, Table 2). An increase in the monomer concentration up to 0.8 M did not afford noticeable amounts of the polymer after 24 h of polymerization. A further increase in the monomer concentration resulted in a dramatic rise in the polymer yield. At the same time, polymer in appreciable yields can still be obtained at low monomer concentrations if the reaction time is significantly increased (Figure 3, square markers).

By varying the monomer concentration and monomer/Pd molar ratio, we succeeded in obtaining high-molecular-weight samples of the addition polymer based on 3-pentafluorophenyltricyclononene-7 (Table 2). The prepared polynorbornene is readily soluble in common organic solvents (Table 3), thus facilitating thin film preparation by a solution casting method onto a cellophane or glass surface. The mechanical properties of poly(3-pentafluorophenyltricyclononene-7) (APF5) are similar to those of other glassy addition polynorbornenes (e.g., the elongation at break and strain strength were 4% and 20 MPa, respectively, for APF5).

**Figure 4:**
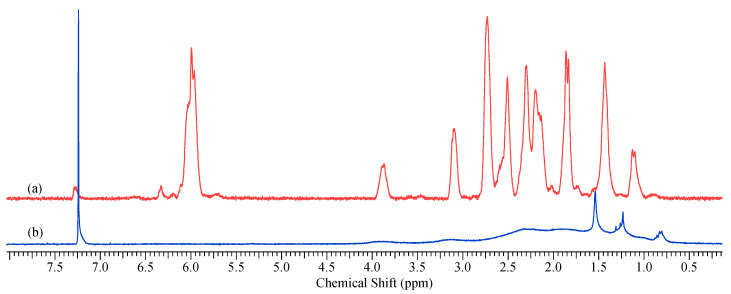
^1^H NMR spectra of (**a**) 3-pentafluorophenyltricyclononene-7 and (**b**) its addition polymer (CDCl_3_).

The structure of the prepared addition poly(3-pentafluorophenyltricyclononene-7) (APF5) was confirmed by ^1^H-, ^13^C-, and ^19^F-NMR spectroscopy and IR spectroscopy. The absence of signals from olefinic protons in the ^1^H NMR spectrum of APF5 clearly evidences the saturated nature of the polymeric main chains (Figure 4).

### 3.2. Physico-Chemical Properties

**Figure 5:**
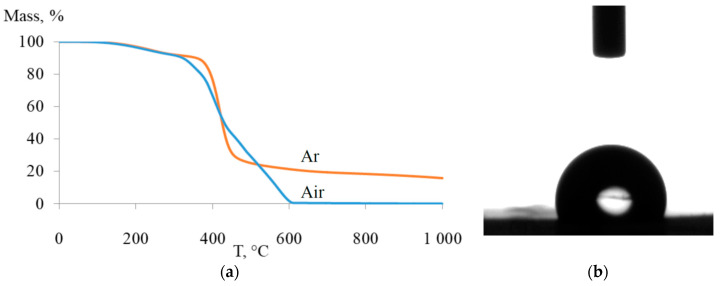
(**a**) TGA curves and (**b**) typical water contact angle for poly(3-pentafluorophenyltricyclononene-7) (APF5).

The synthesized APF5 is a glassy polymer. Its glass transition temperature (T_g_) was not observed by DSC analysis until the beginning of the polymer’s decomposition; the probable T_g_ of APF5 is therefore higher than 300 °C. APF5 exhibited good thermal stability: 5 mass% loss was observed at temperatures higher than 240 °C (Figure 5).

Implementing static contact angle measurements shed light on the hydrophobic nature of the studied polymer. Measurements of the contact angle of APF5 with deionized water resulted in an average value of 101° (Figure 5b). Consequently, the surface of APF5 is hydrophobic with low surface energy.

**Figure 6:**
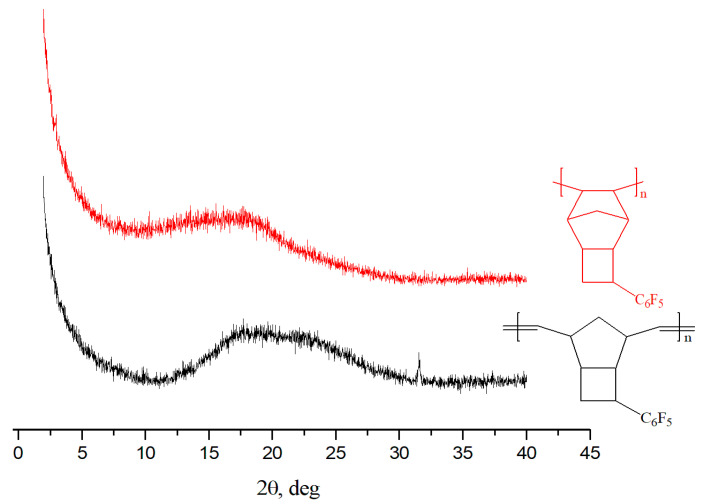
Wide-angle X-ray diffraction (WAXD) patterns for APF5 and MPF5.

Wide-angle X-ray diffraction (WAXD) indicated that APF5, like its metathesis isomer, is completely amorphous (Figure 6). Its WAXD pattern presents one broad peak with a maximum 2*θ* value of 16.1° (Table 4). This differs from the WAXD pattern of the addition polymer derived from unsubstituted norbornene. The interplanar distance calculated with Bragg’s formula (d = λ/2sin *θ*) for APF5 is greater than that for isomeric MPF5, indicating a less dense packing of polymer chains in the case of APF5.

### 3.3. Gas Transport Properties

The gas transport properties of APF5 were investigated using the Daynes–Barrer technique. The permeability (*P*) and diffusivity (*D*) coefficients of different gases (He, H_2_, N_2_, O_2_, CO_2_, and CH_4_) in APF5 were experimentally determined, while the solubility coefficients (*S*) were calculated using Equation (5), and the results are summarized in Table 5. The gas permeability coefficients in APF5 are in the order *P*(CO_2_) > *P*(He) > *P*(H_2_) > *P*(O_2_) > *P*(CH_4_) > *P*(N_2_). The D values for gases in the studied polynorbornene sample decrease as the kinetic diameter of the gas molecule increases: *D*(O_2_) > *D*(CO_2_) > *D*(N_2_) > *D*(CH_4_). As expected, the highest S values for APF5 were observed for the most easily condensable gases—carbon dioxide and methane. As a result, the solubility coefficients of gases in APF5 are in the order *S*(CO_2_) > *S*(CH_4_) > *S*(O_2_) > *S*(N_2_).

**Figure 7:**
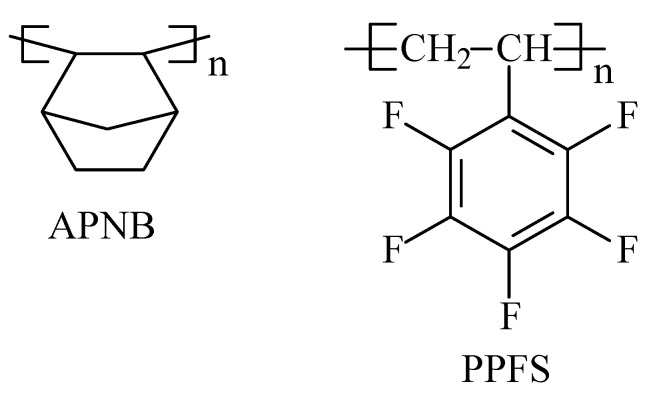
The structures of addition polynorbornenes (APNB) and poly(pentafluorostyrene) (PPFS).

The synthesized APF5 is much more permeable than the similar polymer with C_6_F_5_ side groups attached to linear main chains, poly(pentafluorostyrene) (PPFS, Table 5, Figure 7). The higher gas permeability of APF5 in comparison with PPFS can be explained by a synergetic effect achieved by the presence of both rigid main chains and bulky C_6_F_5_ side groups.

An interesting peculiarity observed for APF5 is the substantially improved gas permeability of APF5 compared to that of addition polynorbornene without side substituents (Figure 7, APNB, e.g., for APNB P(O_2_) ~ 7 Barrer, P(N_2_) ~ 1.5 Barrer [40]). Earlier, it was demonstrated that the incorporation of phenyl rings as side groups in polynorbornenes did not lead to an increase in gas permeability. Moreover, in some cases, it resulted in a decrease in the gas permeability of a polymer, most likely due to π–π stacking of phenyl rings leading to a more dense packing of polymer chains [5,42,43]. The same was found with regards to MPF5: the gas permeabilities of APNB and MPF5 are similar. Eventually, we observed the dramatic difference in gas permeability of APF5 and APNB. Thus, the incorporation of fluoroorganic substituents in addition polynorbornene seems to be an effective tool in the macromolecular design of new productive polymeric membrane materials.

**Figure 8:**
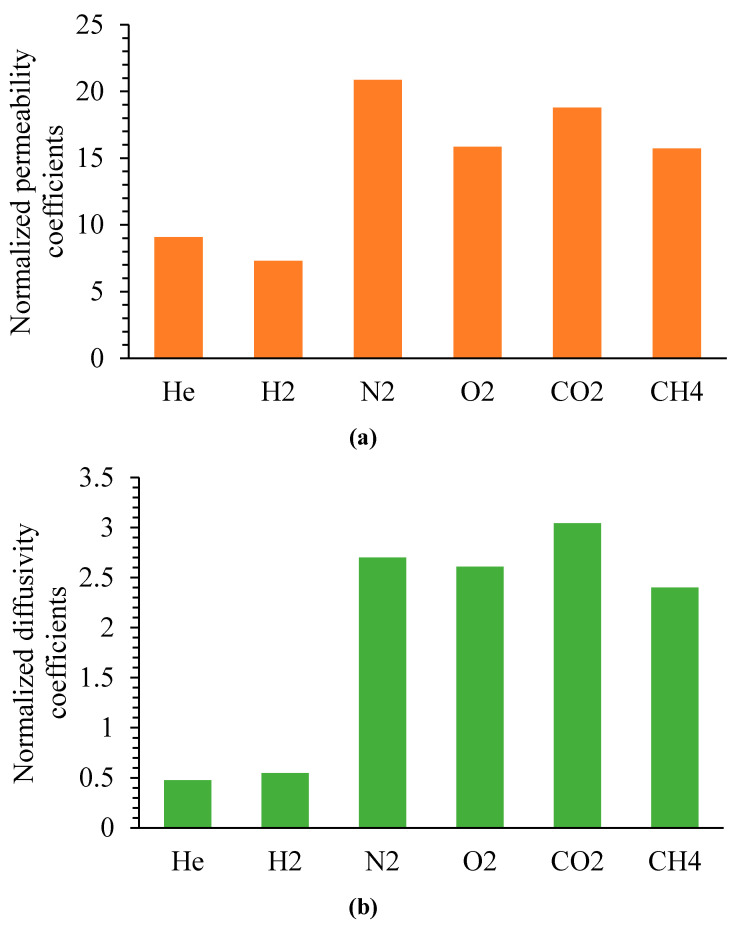

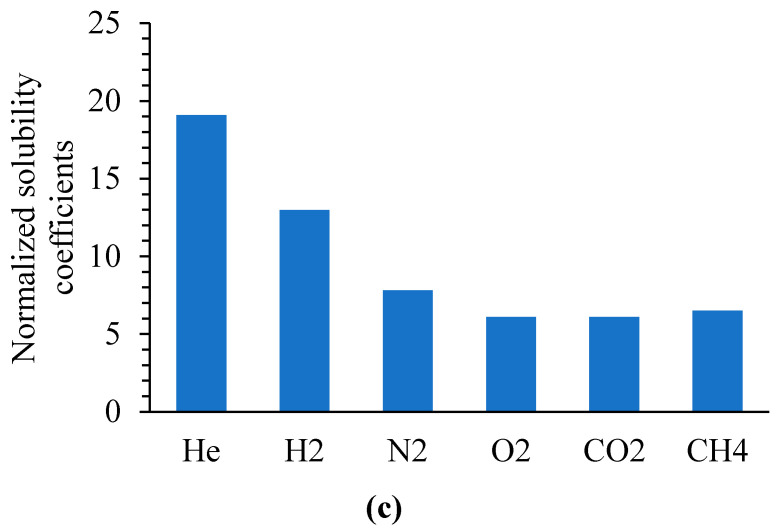
(**a**) Permeability coefficients of gases in APF5 normalized to the permeability coefficients of gases in MPF5; (**b**) diffusivity coefficients of gases in APF5 normalized to the diffusivity coefficients of gases in MPF5; (**c**) solubility coefficients of gases in APF5 normalized to the solubility coefficients of gases in MPF5.

It is of interest to compare the gas transport properties of two isomeric polynorbornenes bearing fluoroorganic side substituents—metathesis (MPF5) and addition (APF5) polymers. Questioning the previously undoubted advantage of addition polynorbornenes in regard to permeation properties, it was recently shown that metathesis polynorbornenes can demonstrate superior permeability when compared to addition isomers. The outcome depends on the nature of the side substituents [25]. Herein, we show, for the first time, that an addition polynorbornene bearing fluoroorganic side groups (APF5) is more permeable than its metathesis isomer (MPF5). The gas permeability of these two polymers differs by more than one order of magnitude (Figure 8). A stronger increase in the permeability coefficients was observed for larger gas molecules (N_2_, O_2_, CO_2_, and CH_4_), while for smaller gas molecules (He and H_2_), the effect was less pronounced. This difference in gas permeability between APF5 and MPF5 can be explained by the loose packing of polymer chains in the case of APF5 that was confirmed by the aforementioned WAXD results. In turn, the looser packing of polymer chains in APF5 is attributed to the rigid main chains of addition polynorbornenes [44]. Analysis of the D and S values for MPF5 and APF5 (Figure 8) showed that the increase in the solubility coefficients made the larger contribution to the observed change in gas permeability. It should be noted that APF5 and MPF5 have close molecular weights, and earlier it was found that the influence of the molecular weights of polynorbornenes on gas transport properties is insignificant in a wide molecular weight range for different gases [45]. The ageing of the membranes from APF5 was not studied in this work, and will be studied later. Nevertheless, it has recently been shown that similar addition polynorbornenes with alkyl or tri(alkoxy)silyl side groups are not prone to ageing for several months [25,29].

The permeability selectivity of separation for APF5 was determined as the ratio of the permeability (P) coefficient of component *i* to that of component *j* (Equation (4)). The obtained results are summarized in Table 6. For APF5, the highest values of selectivities of separations were found for such pairs of gases as CO_2_/N_2_ and CO_2_/CH_4_. These values are close to the ones previously calculated for the less-permeable MPF5 and, therefore, a traditional trade-off between P and α values was not observed. At the same time, in the case of other pairs of gases, the selectivities of separations for APF5 are similar to or lower than those of related, less-permeable polymers, MPF5 and APNB. Unlike the other gas pairs like O_2_/N_2_ and CO_2_/N_2_, the CO_2_/CH_4_ selectivity of APF5 is slightly higher than that of MPF5. This is explained by a larger contribution of diffusivity selectivity (Table 6). The observed difference in the influence of diffusion selectivity on permeability selectivity for APF5 and MPF5 is not completely clear, and this issue can be clarified when a number of similar addition polynorbornenes are synthesized and their properties are investigated.

Using Equation (1), the permeability selectivity of separations was factorized into diffusivity selectivity and solubility selectivity according to Equation (4). The values of α_D_ and α_S_ for APF5 are summarized in Table 6 in comparison with analogous parameters for MPF5. The largest α_D_ values for APF5 were observed for the H_2_/N_2_, H_2_/CH_4_, and He/CH_4_ pairs. Therefore, the contribution of α_D_ to the permeability selectivities for these pairs of gases is major. The values of α_D_ for the H_2_/N_2_, H_2_/CH_4_, and He/CH_4_ pairs in the case of APF5 are lower than those of the less-permeable MPF5. This may be due to the different architecture of the free volume in these polymers. At the same time, α_S_ made the larger contribution to permeability selectivities for pairs of gases containing carbon dioxide and/or methane. This is likely due to the enhanced solubility coefficients of CO_2_ and CH_4_ (Table 5).

## 4. Conclusions

For the first time, the addition polymerization of 3-pentafluorophenyltricyclononene-7 was carried out, and the gas transport properties of the prepared polymer were evaluated. A three-component catalyst system based on palladium acetate, a borate, and tri(cyclohexyl)phosphine was shown to be an effective catalyst for the polymerization of 3-pentafluorophenyltricyclononene-7. This catalyst afforded a soluble vinyl-addition polymer in good or high yields with a high molecular weight and satisfactory film-forming properties. A dramatic influence of the initial monomer concentration on the polymerization process was observed—there is a threshold concentration that enables the polymerization process to proceed efficiently. The synthesized fluorinated addition polynorbornene is a hydrophobic, amorphous, and glassy polymer. The evaluation of the gas transport properties of addition polynorbornene with fluoroorganic side groups was performed for the first time, revealing superior gas transport properties when compared to the isomeric metathesis polynorbornene, as well as to the related polymer with flexible main chains bearing C_6_F_5_ side groups, poly(pentafluorostyrene). The higher gas permeability achieved for addition polynorbornene with C_6_F_5_ side groups was a result of the synergetic effect of the presence of both rigid main chains and bulky C_6_F_5_ side groups. Thus, the incorporation of fluoroorganic substituents in addition polynorbornene seems to be an effective approach in the macromolecular design of new chemically and thermally stable polymers, thus increasing the diversity of materials for membrane gas separation.

## Figures and Tables

**Table 1:** Addition polymerization of 3-pentafluorophenyltricyclononene-7 (the catalyst was Pd(OAc)_2_/PCy_3_/NaBARF, the Pd(OAc)_2_/PCy_3_/NaBARF molar ratio was 1:2:5, [monomer] = 0.7 M, 14 days, the solvent was chloroform, T = 30 °C).

Monomer/Pd Molar Ratio	Yield of the Polymer, %
3000:1	traces
1500:1	11
1000:1	36
500:1	67

**Table 2:** Addition polymerization of 3-pentafluorophenyltricyclononene-7 (the catalyst was Pd(OAc)_2_/PCy_3_/NaBARF, the Pd(OAc)_2_/PCy_3_/NaBARF molar ratio was 1:2:5, [monomer] = 0.7 M, the solvent was chloroform, 24 h, T = 30 °C).

Monomer/Pd Molar Ratio	Monomer Concentration, M	M_w_ × 10^−5^	M_n_ × 10^−5^	M_w_/M_n_
1500:1	1.2	4.10	1.12	3.6
1000:1	0.7	2.93	0.86	3.4
	0.8	3.63	0.95	3.8
	1.2	4.10	1.10	3.7
	1.4	4.35	1.09	4.0

**Table 3:** Solubility of addition and metathesis poly(3-pentafluorophenyltricyclononenes-7) *^a^*.

Solvent	Polymer	
	APF5	MPF5
Dimethyl sulfoxide	-	-
Toluene	+	+
Chloroform	+	+
Tetrahydrofuran	+	+
Dimethyl formamide	-	-
Hexafluorobenzene	+	-
Octafluorotoluene	-	-
Hexane	-	-
1,2,4-Trichlorobenzene	+	+

*^a^* The mark “+” means that the polymer is soluble and the mark “-“ means insoluble.

**Table 4:** The results of WAXD study of some addition and metathesis polynorbornenes.

Polymer	(2*θ*)_1_	d_1_-spacing, Å	(2*θ*)_2_	d_2_-spacing, Å
APNB ^a^ [40]	10.0	8.8	18.5	4.7
MPF5 [34]	18.0	4.9	-	-
APF5	16.1	5.5	-	-

^a^—addition polynorbornene.

**Table 5:** Permeability, diffusivity, and solubility coefficients of various gases in polynorbornenes.

Polymer	Gas						Ref.
	He	H_2_	N_2_	O_2_	CO_2_	CH_4_	
	Permeability (P), Barrer						
PPFS	88	65	5.9	14	50	5.1	[41]
MPF5	44	41	2.3	8.2	33	3.5	[34]
APF5	400	300	48	130	620	55	This work
	Diffusivity coefficients *D* × 10^7^, cm^2^/s						
MPF5	200	53	1.0	2.3	0.92	0.35	[34]
APF5	95	29	2.7	6.0	2.8	0.84	This work
	Solubility coefficients S × 10^4^, cm^3^ (STP)/(cm^3^ × cm Hg)						
MPF5	2.2	7.7	23	36	360	100	[34]
APF5	42	100	180	220	2200	650	This work

**Table 6:** Permeability selectivities for different pairs of gases for polynorbornenes.

Polymer	Pairs of Gases					
	O_2_/N_2_	CO_2_/N_2_	CO_2_/CH_4_	H_2_/N_2_	H_2_/CH_4_	He/CH_4_
	**Permeability Selectivity**					
PPFS [41]	2.4	8.5	10.0	11.0	12.7	17
MPF5 [34]	3.6	14.3	9.4	17.8	11.7	12.6
APF5	2.7	12.9	11.7	6.3	5.5	7.3
	**Diffusivity Selectivity**					
MPF5 [34]	2.3	0.9	2.6	53	151	571
APF5	2.2	1.0	3.3	10.7	34.5	113.1
	**Solubility Selectivity**					
MPF5 [34]	1.6	15.7	3.6	0.33	0.08	0.02
APF5	1.2	12.2	3.4	0.56	0.15	0.06
